# Short-Term Core Strengthening Program Improves Functional Movement Score in Untrained College Students

**DOI:** 10.3390/ijerph17228669

**Published:** 2020-11-22

**Authors:** Tijana Šćepanović, Branka Protić-Gava, Goran Sporiš, Tomislav Rupčić, Zvonko Miljković, Konstantinos Liapikos, Draženka Mačak, Dejan M. Madić, Nebojša Trajković

**Affiliations:** 1 Faculty of Sport and Physical Education, University of Novi Sad, 21000 Novi Sad, Serbia; tijanascepanovic021@gmail.com (T.Š.); brankapg@gmail.com (B.P.-G.); dekimadic@gmail.com (D.M.M.); nele_trajce@yahoo.com (N.T.); 2 Faculty of Kinesiology, University of Zagreb, 10000 Zagreb, Croatia; goran.sporis@kif.unizg.hr (G.S.); tomislav.rupcic@kif.unizg.hr (T.R.); 3 Faculty of Mechanical Engineering, University of Osijek, 35000 Slavonski Brod, Croatia; apartmanmiljkovica@gmail.com; 4 Primary School of Demenika, 26000 Patras, Greece; kliapikos@yahoo.gr

**Keywords:** functional movement screen, injury prevention, core strength, stability training, students

## Abstract

Functional movement is an important part of developing athletes’ but also untrained individuals’ performance. Its monitoring also proved useful in identifying functional limitations and asymmetries, and also in determining the intervention effects. The quasi-experimental pre-test post-test study investigated the effects of core stability training program on the Functional Movement Screen (FMS) score in untrained students after six weeks. The intervention (INT) and control (CG) groups included 73 and 65 male students, respectively. Functional movement patterns were evaluated using the FMS including seven components scores representing seven basic functional patterns. Both groups significantly improved almost all FMS components scores, but the INT increased the mean performance of the hurdle step (partial *ŋ*^2^ × 100 = 4%, *p* = 0.02), in-line lunge (partial *ŋ*^2^ × 100 = 3%, *p* = 0.05), rotatory stability (partial *ŋ*^2^ × 100 = 4%, *p* = 0.02) and total FMS (partial *ŋ*^2^ × 100 = 3%, *p* = 0.04) significantly more than the CG. This justifies that core strengthening can improve FMS in untrained individuals even with the short duration programs.

## 1. Introduction

There is a clear evidence that a sedentary lifestyle, low physical fitness and levels of physical activity represent risk factors for musculoskeletal injuries [1]. Health benefits of physical activities mainly depend on engagement at recommended levels [2]. Accordingly, there are great number of activity-induced injuries among young adults [3,4].

Despite possible limitations in determining the risk factors for injuries, some screening measures have demonstrated promise in various populations [5]. Traditional tests for evaluation of strength or range of motion cannot detect fundamental changes in motor control [6]. However, the Functional Movement Screen (FMS) aims to identify imbalances in mobility and stability during seven fundamental movement patterns [7]. Although the FMS is primarily designed to screen active adults for future injury, determining a baseline of movement competence could allow comparisons after treatment or rehabilitation [5].

Previous studies have examined the effects of various training interventions on the FMS in different groups of participants where short-term functional movement training was not enough to improve the FMS performance in adolescents [8]. Additionally, individually based specific mobility and neuromuscular control training did not elicit positive changes in the FMS scores in firefighters after 12 weeks [9]. However, the improvements in the FMS scores were shown after a six-weeks training program in children [10], a yoga program [11], and a six-week functional training program [12]. At higher competitive levels in professional American football players, the improvements in the FMS scores were also collected after a seven-week corrective exercise program [6].

Core stability has been considered as a decisive factor in foundation for movement of the extremities, for supporting loads, and for the protection of the spinal cord and nerve roots [13]. It has also been speculated that inadequate static core stability may compromise the dynamic stability of the extremities, which could lead to increased stress on the soft tissue [14,15], and the appearance of repetitive strain injuries [16].

Improved postural stability and increased core endurance has been shown in university students following an eight-week thoracic spine stabilization exercise program [17]. It has been stated that core training emphasizes strength and conditioning of the local and global muscles that work together to stabilize the spine [16]. Accordingly, a significant improvement was found in the back-endurance tests after a 10-week stability ball core training program [18]. Similarly, positive effects from a spinal stability exercise protocol using Swiss balls was winkled out in sedentary people [19].

The benefits of FMS in injury prediction have been documented [5]. However, injuries are not just characteristic of athletes. Therefore, identifying weaknesses in healthy adults and then trying to improve it could play a significant role for lifelong physical activity and movement [20]. Moreover, it was demonstrated recently that FMS performance has a strong association with key health markers [21]. Therefore, the aim of the present study was to investigate the effects of core stability training program on the FMS scores in young untrained adults. It was hypothesized that core strengthening training would improve the FMS of students.

## 2. Materials and Methods

A quasi-experimental, pre-test/post-test control group design was used in this study. University students from Novi Sad, Serbia volunteered to participate and were placed into one of two groups: intervention and control group. The intervention consisted of the implementation of a core exercise program three times per week. The FMS scores were examined before and after a six-week training intervention. Participants were tested for changes in basic patterns of movement that were measured by Cook’s FMS [7]. All participants were asked to continue their standard obligations at the faculty and to refrain from making any changes to their physical activity habits for the six-week period.

### 2.1. Participants

**Figure 1:**
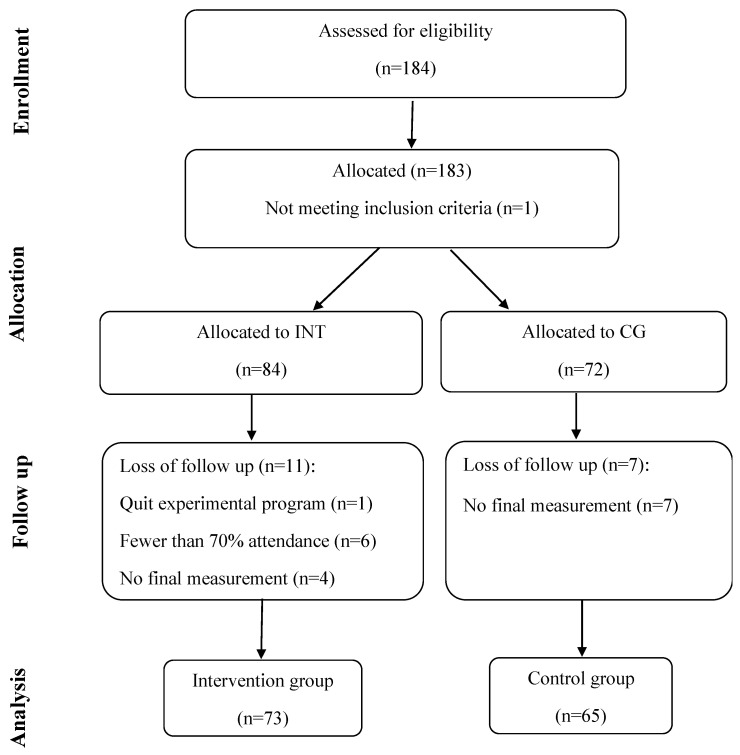
Flow diagram of participant enrolment, group allocation and final analysis.

A total of 138 males were divided into two groups, intervention (INT) (mean ± SD: n = 73, age 20 ± 0.5 years, height 180 ± 5.20 cm, body mass 76 ± 9.4 kg), and control (CG) (n = 65, age 20 ± 0.7 years, height 181 ± 8.10 cm, body mass 78.6 ± 4.7 kg). We included healthy subjects who were not engaged in any systematic training in the last two years, but they had systematically practiced a sport for at least three years. Participants were free of any musculoskeletal injury or illness. University ethics board approved the study (ref. no. 20/2018), and all the participants gave their informed consent before any data collection. Testing procedures were performed following the ethical standards laid down in the Declaration of Helsinki. Figure 1 presents flow diagram for study participants.

### 2.2. Testing Procedures

Functional movement patterns were evaluated using the FMS [22]. The FMS identifies limits in seven basic functional patterns: deep squat, hurdle step, in-line lunge, shoulder mobility, active straight leg raises, trunk stability push-up and rotary stability. Reliability of FMS was confirmed in previous studies [23,24].

Testing was performed using a model of score from 0–3 [22]. The zero score was given to the respondents who felt pain during the tests. In cases when they felt pain, regardless if the task had been executed or not, they did not pass the test. Score one was given to the respondents who did not do the test by following given instructions. Score two indicated that the respondent could carry out the given movement, but there was a lower degree of limitation or compensation for the movement. Score three was given to the respondents who performed the movement in the described way, without any compensation. The overall score of the screening represented the sum of individual assessments of each test, totaling 21. Five of seven tests were bilaterally tested, meaning both for the left and right body side individually. In the case of bilateral tests, when the respondent received a lower score for one body side, that score was taken as an overall score for that specific test.

Initial testing was conducted in October and the final testing was conducted in December. All subjects were tested during the semester, with subject testing occurring at the start of the day, just before the lessons. A familiarization day supervised by a certified strength and conditioning specialist was implemented a week prior to conducting the baseline tests to ensure that the participants understood the testing procedures and to demonstrate reliability in testing measures.

The FMS was recorded using two video cameras (Panasonic, NV-GS400, Panasonic Corp., Osaka, Japan) placed in the frontal and sagittal planes and scored later. Two raters, both of whom had two years of experience using the FMS in clinical practice, scored participant performance on the movement tasks. A subgroup analysis showed good interrater reliability between the raters for composite scoring (intraclass correlation coefficient, 0.897).

### 2.3. Program

Participants were required to take part in a six-week program which included supervised sessions three times per week with sessions lasting 30 min each. Core strengthening program involved an isometric exercise program, sustained contraction against an immovable load or resistance with no or minimal change in length of the involved muscle group [10] and was implemented in the first and second week. The exercises were focused on the weakest and asymmetrical scores, with primary focus on mobility patterns and secondary focus onto stability patterns. Most of the exercises included activities where the upper extremity and the lower extremity were active at the same time while holding a neutral abdominal posture. Exercises of anti-flexion, anti-extension and anti-rotation were used to stabilize the spine. The treatment included exercises for increasing the mobility and strength of the neck, shoulders, pelvis and hips. In the first two weeks, participants did various durability exercises (all position planks, plank walk, plank jacks, forearm to pushup plank, etc.). The third and fourth week included similar exercises reinforced with additional movements and rotations (alternating arm and leg plank hold, superman plank hold, front plank plate switches, etc.). During the fifth and sixth week, the exercises were performed on unstable surfaces (Table 1). The McGill curl up [25], performed by flexing torso in the supine position with elbows on the floor, was performed during every treatment for all six weeks.

Both groups were not involved in any systematic exercise training except a regular program at the Faculty of Sports. The program represents a group of activities carried out at the Faculty of Sports during the second year. The sports activities represented in the regular classes were football, handball and rhythmic gymnastics. Respondents learned only techniques in the listed sports.

### 2.4. Statistical Analysis

Data were analyzed using SPSS (version 20.0, IBM Corp., Armonk, NY, USA). Mean and standard deviations for the pre- and post-test FMS scores were calculated for each group. Log-transformed data were analyzed if a Kolmogorov-Smirnov test rejected normality but original data were reported for the sake of clarity. The Levene’s and Box’s tests failed to reject homogeneity of variances and covariance matrices, respectively. 

A 2 (INT vs. CG) × 2 (baseline and after six weeks) mixed-model ANOVA evaluated the intervention effects on the FMS test outcomes, and a group-by-time interaction effect (a 2 × 2 interaction effects) was the hypothesis of primary interest. We analyzed the simple main effects of time to show the mean changes in the FMS scores after six weeks for each group, and the six-week-induced changes are reported as percentage of change (% Δ). Partial eta squared (partial *ŋ*^2^) is reported as a measure of an effect size for the interaction effects, and defined as small (0.01), medium (0.06), and large (0.14) [26]. Bonferroni adjusted *p*-values for multiple comparisons, and the level of significance was set at *p* ≤ 0.05.

## 3. Results

After six weeks, the INT and CG mean performance significantly improved in six and five FMS component tests, respectively. Both groups also enhanced total FMS score after six weeks. However, the INT increased the mean performance of the hurdle step, in-line lunge, rotatory stability, and total FMS to a significantly greater extent as compared to the CG. For detailed results of the 2 × 2 mixed-design ANOVA model, see Table 2.

## 4. Discussion

There is currently very little evidence of the ability to change scores on the FMS following a core strength training program. Accordingly, this study aimed to examine the effects of core strength training on FMS scores in university students. The results of this study showed that both groups improved significantly in the overall score on FMS. Previous research indicates that the average FMS scores are highest within the 20–39 age group (mean: 15.08), and lowest for those who were age 65 years (12.68) [20]. At pre-test, participants in our study had higher values for the total FMS score (mean ± SD: intervention group 16.49 ± 1.28, control group 16.29 ± 1.52) compared to those norms. Therefore, it can be speculated that improvements would be even better with lower initial values for FMS. 

Individually analyzing variables, the positive effects of the intervention are visible in the tests: hurdle step (*η*_p_^2^ = 0.04), in-line lunge (*η*_p_^2^ = 0.03), rotatory stability (*η*_p_^2^ = 0.04), total FMS (*η*_p_^2^ = 0.03). Core stability is of great importance in all three individual tests. In the hurdle step and in-line lunge tests, the support is on the standing leg while the movements are performed at the hip, knee and ankle. On that occasion, the fixators play an important role in order to stay upright without losing balance. It is similar when performing the rotator stability test. The FMS is used to evaluate performance with fundamental movements and to find deficits in the body during dynamic movements [7,22]. Accordingly, the core becomes activated before gross body movements as part of the postural control system [16,27]. The current study used a variety of core-stabilization training methods that included muscle endurance exercises, exercise using unstable surfaces, and performing some exercises in a weightbearing position, which may be the reason why scores in the FMS improved.

In summary, the main idea was to develop core stability throughout core muscle isometric strengthening, because ideal quality of core stabilization is a foundation for any proper movement, and then progress to dynamic stability exercises by incorporating limb movements and changing positions [16]. In general, the core stability affects the effective use of the strength and endurance required [28].

This study supports the use of exercise interventions in university students for the improvement in the FMS score. There is sufficient evidence for the effectiveness of core stability training in untrained individuals. Functional movement screen test results showed greater change for the exercise group compared with the control group. This result justifies the hypothesis that core strengthening can improve FMS in college students even with short duration programs. Similar results were obtained by applying a periodized functional strength training program to the total FMS score in college students of physical education [29].

We had some limitations, of which the most important could be listed as the nonexistence of randomization. The study did not include effects on other motor skills. High baseline values also significantly contributed to a smaller effect size in our study. 

## 5. Conclusions

Relative to research findings, it is safe to infer that FMS could be used for the assessment of changes in movement pattern induced by six-week core-stabilization-training. Isometric core strengthening with an exercise program involving multiplane movements has plausible benefits to functional movement patterns. Practitioners working in this population should consider the specific changes in the intervention group in this study. It would be interesting for future studies to consider the long-term effects of core strengthening program, as well as the application of programs for people with existing pain syndromes.

## Figures and Tables

**Table 1:** Core strengthening program.

Week	Exercises	Sets, Reps, Time
1–2	All position planks (pushup, forearm, side, reverse)	Hold (average) 50 s
	Plank walk	
	Plank jacks	
	Forearm to pushup plank	
	Feet elevated side plank	
	3-point plank	
	Bird dog	
3–4	Alternating arm plank hold	20–30 reps
	Alternating leg plank hold	
	Superman plank hold	
	Side plank star hold	
	Alligator plank walk	
	Plank barrel roll	
	Bird dog	
5–6	Plank down dog to toe tap	20–30 reps
	Plank with single arm fly, feet on ball	
	Feet elevated side plank	
	Side plank hip adduction circle	
	Front plank plate switches	
	Side plank raises on ball	
	Swiss-ball stir the pot	
	Bird dog	
1–6	McGill curl up	20 reps

**Table 2:** The Functional Movement Screen (FMS) scores at pre- and post-test for the intervention and control group.

Outcome	Pre-Test	Post-Test	% Δ	A Group-by-Time Interaction Effect			
Group	Mean ± SD	Mean ± SD		F _(1, 136)_	*p*	Partial *ŋ*^2^	1-β
Deep Squat (score)							
INT	2.32 ± 0.57	2.52 ± 0.50	+8.6 **	0.51	0.48	0.00	0.11
CG	2.32 ± 0.64	2.51 ± 0.59	+8.2 *				
Hurdle Step (score)							
INT	2.05 ± 0.28	2.32 ± 0.50	+13.2 **	5.48	0.02	0.04	0.64
CG	2.08 ± 0.32	2.15 ± 0.40	+3.4				
In-line Lunge (score)							
INT	2.18 ± 0.54	2.56 ± 0.53	+17.4 **	3.77	0.05	0.03	0.49
CG	2.22 ± 0.57	2.40 ± 0.58	+8.1 *				
Shoulder Mobility (score)							
INT	2.84 ± 0.37	2.89 ± 0.31	+1.8	2.27	0.14	0.02	0.32
CG	2.48 ± 0.64	2.63 ± 0.51	+5.7 **				
Active Straight Leg Raise (score)							
INT	2.49 ± 0.60	2.68 ± 0.49	+7.6 *	1.29	0.23	0.01	0.20
CG	2.51 ± 0.64	2.80 ± 0.40	+10.4 **				
Trunk Stability Push Up (score)							
INT	2.63 ± 0.49	2.89 ± 0.32	+9.9 **	3.25	0.07	0.02	0.43
CG	2.71 ± 0.49	2.83 ± 0.38	+4.4 *				
Rotatory Stability (score)							
INT	1.96 ± 0.20	2.15±0.36	+9.7 **	6.13	0.02	0.04	0.69
CG	1.98 ± 0.33	2.03 ± 0.17	+2.5				
Total FMS (score)							
INT	16.45 ± 1.27	18.01 ± 1.57	+9.5 **	4.18	0.04	0.03	0.53
CG	16.31 ± 1.51	17.43 ± 1.47	+6.9 **				

Values are Mean ± SD; Abbreviations: INT intervention group; CG control group; % Δ percentage of change from pre- to post-test; F(dffactor, dferror) = F-statistic; p probability value; partial *ŋ*^2^ partial eta squared; ** significant changes after six weeks at *p* < 0.001; * significant changes after six weeks at *p* ≤ 0.05.
